# Validation of Finite Element Model by Smart Aggregate-Based Stress Monitoring

**DOI:** 10.3390/s18114062

**Published:** 2018-11-21

**Authors:** Haibin Zhang, Shuang Hou, Jinping Ou

**Affiliations:** 1 Shenzhen Graduate School, Harbin Institute of Technology, Shenzhen 518055, China; oujinping@hit.edu.cn; 2 Key Lab of Structures Dynamic Behavior and Control of the Ministry of Education, Harbin Institute of Technology, Harbin 150090, China; 3 School of Civil & Transportation Engineering, South China University of Technology, Guangzhou 510641, China; cthous@scut.edu.cn

**Keywords:** RC structure, smart aggregate, stress monitoring, compressive strength, OpenSEES, embedded sensor

## Abstract

Concrete compressive strength is an important parameter of material properties for assessing seismic performance of reinforced concrete (RC) structures, which has a certain level of uncertainty due to its inherent variability. In this paper, the method of concrete strength validation of finite element model using smart aggregate (SA)-based stress monitoring is proposed. The FE model was established using Open System for Earthquake Engineering Simulation (OpenSEES) platform. The concrete strengths obtained from the material test, peak stress of SA, and estimated concrete strength based on SA stress were employed in FE models. The lateral displacement monitored by Liner variable differential transformer and vertical axial load monitored by load cell in the experiment are applied in the model. By comparing the global response (i.e., lateral reaction force and hysteretic loop), local response (i.e., concrete stress, rebar strain, and cross-section moment) and corresponding root-mean-square error obtained from experiment and numerical analysis, the capabilities of validation of FE model using SA-based stress monitoring method were demonstrated.

## 1. Introduction

The assessment of seismic performance of the existing infrastructures is a matter of high priority in earthquake prone areas. Quantifying the material properties of these structures is critical due to the impact that it has on the subsequent application of assessment methods. For reinforced concrete (RC) buildings, concrete compressive strength has emerged as an important material property that requires careful consideration because it directly relates to durability and strength of RC structure.

According to American concrete design code [1], the average compressive strength of concrete (*f_cr_*) obtained from the material test must be higher than the specified compressive strength, *f_c_*, to guarantee the safety of structures. A similar requirement also can be found in Chinese concrete design code [2]. Concrete compressive strength has intrinsic variability, which is usually considered as being a random variable with a certain level of uncertainty [3]. In practice, the *f_cr_* depends significantly on workmanship including mix, casting and curing operations [4,5,6]. Furthermore, the extreme factors during operation stage of concrete structure, such as degradation of the material induced by freezing and thawing [7], chemical attack [8] and fire hazard [9], may result in the actual *f_cr_* being lower than *f_c_*. It has been proven that the coefficient of variation (CV) of concrete compressive strength often exceeds 20% [10]. Therefore, it is necessary to estimate the *f_cr_* of existing structures to establish the suitable FE model for evaluating the seismic performance of RC structure in advance in case of earthquake.

Non-destructive evaluation (NDT) provides a promising way towards *f_cr_* prediction because many NDT parameters are sensitive to concrete strength variations. Two major NDT methods are mostly considered: hardness tests [11,12,13,14] and ultrasonic pulse velocity (UPV) method [15,16,17]. The hardness test can be conducted using rebound hammer based on the principle that the rebound of an elastic mass depends on the hardness of concrete surface against which the mass impinges [18]. However, this method only reflects limited depth close to the concrete surface [19]. Besides, the near surface properties of the concrete such as smoothness, carbonation, size and type of aggregate will significantly affect the test results [13,20]. The expected error of in situ strength prediction with rebound hammer can reach ±30% [14]. In general, UPV measurement are based on the use of a pair of external piezoelectric transducers (i.e., transmitter and receiver) held on the surface of concrete to measure the pulse velocity along the transmitting path [15]. However, this method faces the problem of the unstable coupling between external transducer and concrete surface [21]. Additionally, at least one of the surfaces of the concrete under test should be accessible for transducer deployment. However, most structures need to be inspected in service, which will also have an extra cost for removal of any type of decoration or stop the normal service [22]. To avoid the disadvantages of externally mounted sensors, a promising technique using piezoelectric transducers embedded in concrete structures has been given wide attention [22,23,24,25].

Smart aggregate (SA), as a piezoelectric-based embeddable transducer, is made of a waterproof material packaged PZT patch between concrete or granite blocks to protect it from moisture and fragile broken [25,26]. SA is a multi-functional transducer, which can act as both actuator that generates ultrasonic stress waves and sensor that senses the waves or forces. The result of UPV test using embedded SAs shows good agreement with that using external transducers [23]. Besides velocity, the amplitude of harmonic response was successfully used for evaluating the early-age concrete compressive strength [27,28,29]. The applications of a pair of SAs based on a transmitter–receiver configuration can also be found in structural health monitoring (SHM) of RC structures, such as crack monitoring [24,30,31], damage detection [32,33,34,35] and water seepage monitoring [36,37,38]. Based on sensing configuration, a single SA can also be used for SHM of concrete structures, such as overheight vehicle–bridge collision monitoring [39], embedded acoustic emission damage detection [21], and seismic stress monitoring [26,40,41,42,43,44,45]. Since concrete compressive strength is a characteristic value of stress, theoretically, it can be obtained by using the stress-history that SA monitored if the concrete stress reaches peak stress. However, the report of concrete compressive strength evaluation based on sensing configuration has not been found. Compared to the transmitter–receiver configuration, the sensing configuration of SA takes advantage of direct monitoring, reduction of the instruments to generate the high power ultrasonic wave, and low cost.

In this paper, we report on a feasibility study of concrete compressive strength validation of FE model using SA-based stress monitoring method. The FE model was established through the platform of Open System for Earthquake Engineering Simulation (OpenSEES). The global and local response, and corresponding root-mean-square error were compared.

## 2. SA Sensor

**Figure 1:**
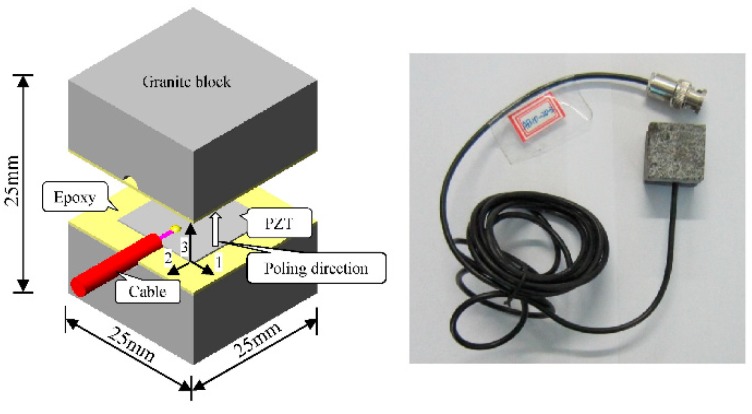
Illustration and photo of the SA.

The SA was fabricated by sandwiching a PZT patch between a pair of granite blocks with epoxy, as illustrated in Figure 1. The dimensions of SA and PZT are 25 mm × 25 mm × 25 mm and 15 mm × 15 mm × 0.3 mm, respectively. The details of material properties are reported in the literature [26]. The SA has been proved to have the potential to monitor the exact stress in concrete structure [26,41].

## 3. Simulation of the RC Column Test

### 3.1. Description of the Finite Element Model

**Figure 2:**
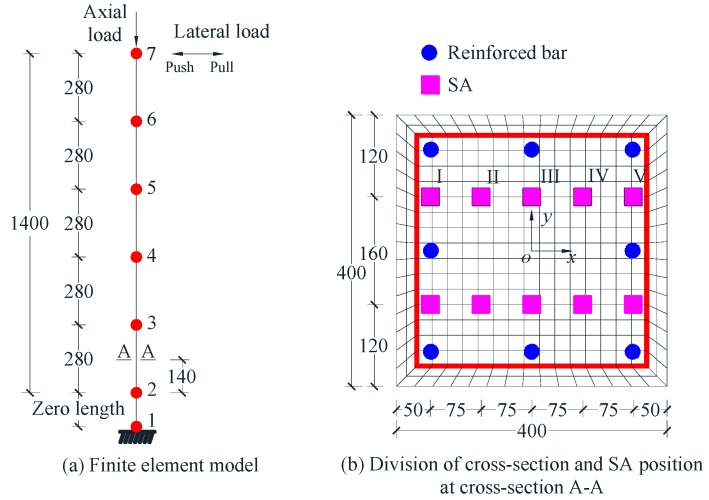
(**a**) Finite element model; and (**b**) division of cross section and SA position of RC column.

The two-dimensional FE model was established. The layout of nodes and elements of the FE model is shown in Figure 2a. A zero-length section element located at the bottom of the beam-column element was employed to consider the bond-slip between longitudinal steel bar and concrete [46], which consists of a pair of nodes (Nodes 1 and 2) with the same vertical and lateral translational degree of freedom. The footing of the column was assumed as rigid due to its relatively large size and stiffness. Since the footing is rigid, it will not influence the deformation of the column and was neglected in the modeling. The other parts of RC column between Nodes 2 and 7 were divided into five elements and modeled using displacement-based nonlinear beam-column element with five Gauss–Legendre integration points. The displacement-based nonlinear beam-column element, implemented in OpenSEES, accounts for linear and nonlinear flexural deformations by assuming plane section at each integration point and capturing the spread of plasticity along the element.

The division of the cross-section at the integration point (140 mm away from Node 2) is presented in Figure 2b. In this cross-section, stresses of the fiber at ten locations that have the same coordinate with SAs were recorded. Since the stresses of two locations with opposite y coordinate and the same x coordinate are equivalent, herein only five locations with passive y coordinate along the loading direction donated as I–V were employed to compare with experimental results. All the divisions of the cross-sections of the column are the same. P-Δ effect of the column was taken into account in the model.

### 3.2. Section and Material Properties

**Figure 3:**
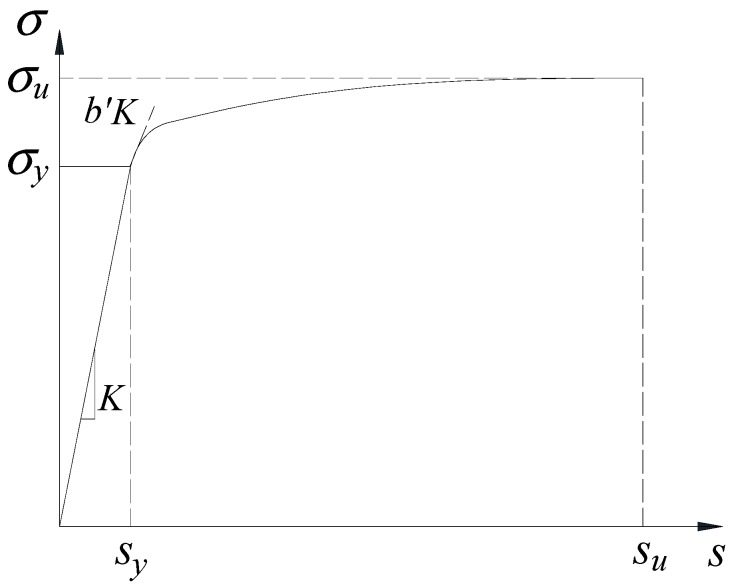
Envelope curve for rebar stress versus loaded-end slip relationship.

The material property of the reinforcement fibers in the zero-length section element proposed in the literature [46] is shown in Figure 3. *σ_u_* is the ultimate strength of the rebar, which is 1.3 times of yield strength *σ_y_*. *s_y_* and *s_u_* are loaded-end slips corresponding to the bar stresses of *σ_y_* and *σ_u_*, respectively. *s_y_* can be calculated using Equation (1) [46]

(1) $$s_{y}=2.54{\left(\frac{d_{b}}{8437}\frac{\sigma_{y}}{\sqrt{f_{c}}}(2\alpha +1)\right)}^{1/\alpha }+0.34\;$$

where *α* is the parameter used in the local bond-slip relation, which is taken as 0.4 in this paper in accordance with provision 6.1 of CEB-FIP Model code 2010 [47]. *d_b_* is the diameter of the longitudinal steel bar, which is 22 mm in this paper. *σ_y_* is the yield strength of the rebar, which is tested to be 460 MPa. *s_u_* = 30–40 *s_y_*, which is set to be 35. As shown in Figure 3, the stiffness reduction factor that defines the stiffness degradation after the rebar yielded ranges 0.3–0.5, and is *b*’ = 0.3 in this study. To employ the model for capturing the pinching characteristic effect of reinforcement in flexural members subjected to reversed cyclic loading, the coefficient *R_c_*, which defines the pinching characteristic, is set to 0.6 according to the literature [46].

**Figure 4:**
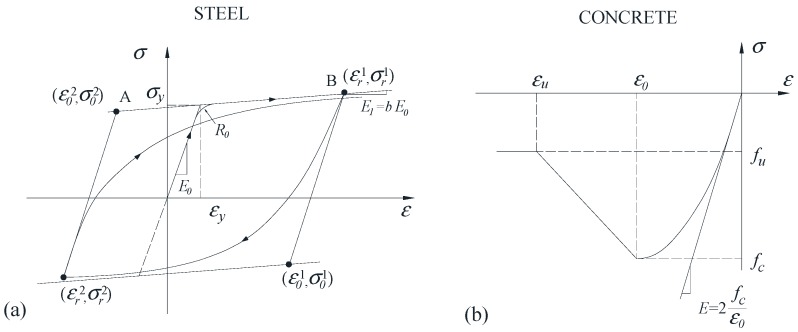
Material constitutive models: (**a**) steel; and (**b**) concrete.

As shown in Figure 4a, the reinforced steel bar follows the nonlinear model of Giuffré–Menegotto–Pinto [48], as modified by Filippou et al. [49] to include isotropic-hardening effects. The Young’s modulus of reinforced steel bar, *E_0_*, is 210 GPa. The yield strength of the rebar σ*_y_* is tested to be 460 MPa, corresponding to the yield strain *ε_y_* of 2190 *με*. The strain-hardening ratio *b* is 0.02. The parameter *R_0_*, which controls the curvature of the transition from the elastic to plastic-hardening branch, is 20 [49].

To further improve the efficiency of simulation and adequately capture the major behavior of concrete in compression rather than tension, the constitutive relationship for the concrete is modeled using the Kent–Scott–Park model [50] with no tension stiffening, as presented in Figure 4b, in which *f_u_* is the ultimate residual strength, *ε_0_* is the strain at peak strength, *ε_u_* is the strain when the residual strength is reached, and *E* is the Young’s modulus of the concrete.

### 3.3. Concrete Strength Selection

It should be noted that *f_c_* is a statistical parameter, which is a variable for the concrete structures. To identify the average concrete compressive strength, four typical parameters, namely lower limit and average value of concrete compressive strength of concrete cube, the peak stress monitored by SA, and the estimated strength based on concrete stress distribution assumption, were selected as the potential concrete compressive strength in the models and denoted FEA-1–4, respectively.

The average strength of the concrete cube specimen cast with the column was 33.5 MPa with a coefficient of variation of 0.13. The unconfined concrete strength of the RC column is considered to be 0.79 times smaller than that of the concrete cube due to end friction effects [51], which is 26.5 ± 3.5 MPa. The relationship between confined and unconfined concrete compressive strength is expressed as follows:

(2) $$f_{cc}=kf_{c}$$

where *f_cc_* is the confined concrete compressive strength induced by stirrups. *κ* is the ratio between *f_cc_* and *f_c_*, which can be expressed as [52]:

(3) $$\kappa =1+\frac{\rho_{s}f_{yh}}{f_{c}}$$

In this paper, the stirrup is plain bars at 100 mm spacing with a diameter of 10 mm. The volume–stirrup ratio, *ρ_s_*, is 0.0092. The yield strength of stirrup, *f_yh_*, is 300 MPa.

The lower limit and average value of concrete compressive strength of concrete cube are considered as the candidates of the unconfined concrete compressive strength of RC column, which were 23.0 and 26.5 MPa, respectively. The corresponding lower limit and average value of the confined concrete compressive strength can be calculated using Equation (2), which are 26.0 and 29.7 MPa, respectively.

The monitored peak compressive stress of SA in experiment is 39.4 MPa [53], which is larger than the upper limit of the concrete compressive strength calculated using the concrete cube specimen, and is considered as one of the potential confined concrete compressive strengths. The upper limit of the unconfined concrete compressive strength is 36.5 MPa, which can be obtained from Equation (2) as well.

**Figure 5:**
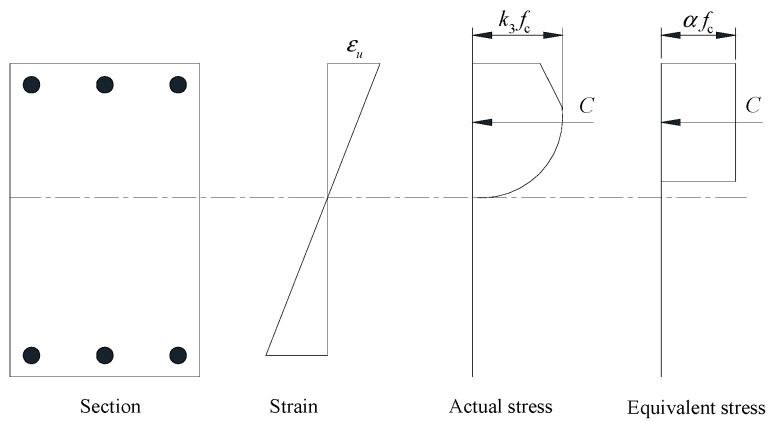
Concrete stress distribution and rectangular stress block.

The stress monitored by SA can be recognized as the average compressive stress of limited zone. Based on the stress distribution assumption, as illustrated in Figure 5, the ratio of concrete compressive strength to average compressive stress is equal to *k_3_*/*α*, where *k_3_* is 0.925 and *α* is 0.85 for normal strength concrete [54]. The estimated concrete compressive strength can be calculated using peak stress of SA and *k_3_*/*α*, which is 42.9 MPa for confined concrete. Utilizing Equation (2), the estimated unconfined concrete can be obtained, which is 40.0 MPa.

The detailed of the concrete constitutive parameters are shown in Table 1. *f_u_* is 0.2 and 0.8 times *f_c_* for unconfined and confined concrete, respectively. In accordance with Chinese code for design of concrete structures [2], the peak strain of unconfined concrete, *ε_0u_* and the ratio of the peak strain to ultimate stain of the unconfined concrete, ε*_0u_*/*ε_uu_*, can be obtained. The ratio of peak strain of confined concrete to that of unconfined concrete, ε*_0c_*/*ε_0u_*, is equal to *ε* as well, which can be obtained using Equation (3). The confined ultimate strain, ε*_uc_*, is a constant, 0.02 in this study.

### 3.4. Loading Scheme

**Figure 6:**
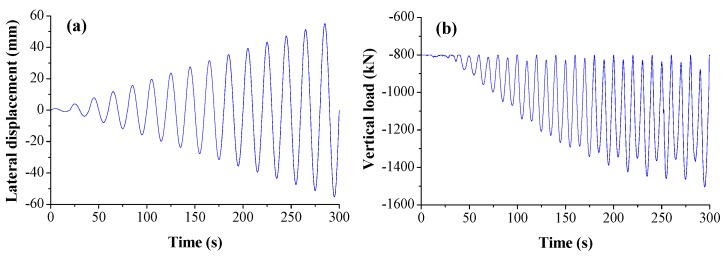
Loading scheme for: (**a**) lateral direction; and (**b**) vertical direction.

The loading scheme for FE analysis was the same as that of the experiment in [53]. The displacement-controlled lateral displacement with a sine harmonic form and load-controlled vertical load with a variation of amplitude were applied on the top of the FE model, respectively, as shown in Figure 6a,b.

## 4. Results and Discussion

### 4.1. Global Response

The global response including lateral load and hysteretic loop were used to evaluate the effectiveness of strength prediction by comparing the results of experiment with that of FE analysis.

#### 4.1.1. Lateral Load

**Figure 7:**
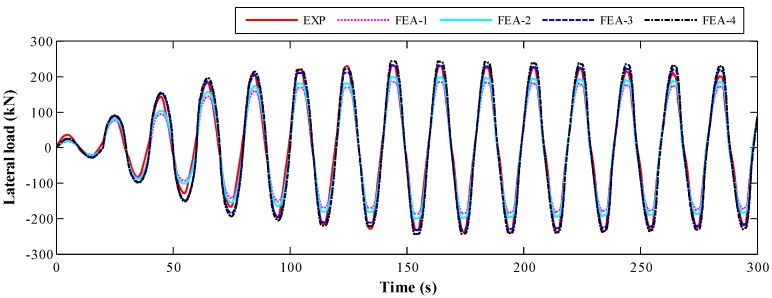
The time-history of the lateral load.

As shown in Figure 7, both the period and variation trend of the lateral load in experiment agree well with that of FE analysis results. With the increase of loading time, the amplitude of each cycle of the curves firstly increased, and then gradually decreased, which reflects the damage process of the specimen until the rebar yield. The curves obtained from FEA-3 that used peak stress of SA and FEA-4 that used estimated strength are almost the same, which is much closer to the experimental results than the FE analysis results that use the concrete cube strength. The experimental peak load was about 250 kN at the eighth cycle, which agrees well with the results of FEA-3 and FEA-4 model.

#### 4.1.2. Hysteretic Loop

**Figure 8:**
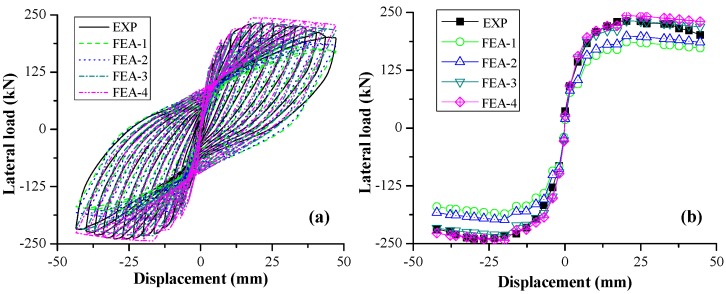
Hysteretic responses of the specimens: (**a**) hysteretic loops; and (**b**) envelop curves.

The lateral load-displacement hysteretic loops are shown Figure 8a. The results of FEA-3 and FEA-4 are similar, which are much closer to experimental curve than those of FEA-1 and FEA-2. The hysteresis performance of FEA-3 and FEA-4 in terms of reloading stiffness, unloading stiffness, and energy consumption are in good agreement with experimental result.

Figure 8b shows the comparison of envelop curves of RC columns between FE analysis and experiment. It was found that the initial stiffness increased with the increase of *f_c_*, which is reasonable based on the relationship between Young’s modulus and *f_c_*, as illustrated in Figure 4b. When the peak load and 85% of peak load were reached, the lateral displacement was about 20 mm and 44 mm, respectively, corresponding to a story drift of 1.43% and 3.14%.

### 4.2. Local Response

Generally, the global response rather than the local response (i.e., concrete stress and rebar strain) was used for evaluating the difference between experimental and FE results, due to the lack of effective local stress and strain monitoring methods, especially in large deformation stage. In this study, utilizing the SAs combined with strain gauge, the local response including concrete stress, rebar strain, and cross-section moment were obtained, providing a new index to evaluate the performance of strength prediction.

#### 4.2.1. Concrete Stress

**Figure 9:**
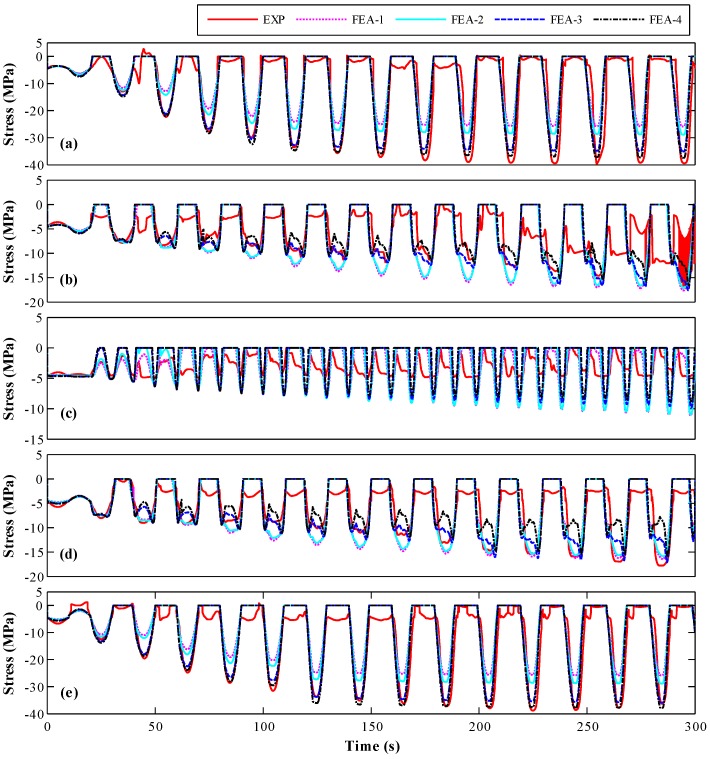
Stress time-history for experiment and finite element analysis at: (**a**) Point I; (**b**) Point II; (**c**) Point III; (**d**) Point IV; and (**e**) Point V.

The stress-histories of five typical locations, as presented in Figure 2b, obtained from experiment and FE models are compared in Figure 9. It is noted that the models FEA-3 and FEA-4 provided a good prediction of the observed response and captured the trend of the stress time-histories. The amplitude of the stress at the locations (Points I and V) away from the center of the cross-section was larger than that of Points II–IV, which are close to the center of the cross-section, due to the larger bending moment. The stress time-history of the SA at the central axial of the cross-section (Point III) is very complicated, which results from the combined effects of the variation of axial load and transformation of neutral axial. As expected, the phase of SAs located at the symmetry positions along the central axial of the cross-section are about 180°. The stress time-histories obtained from FEA-3 and FEA-4 model are similar, which has better agreement with experimental results than FEA-1 and FEA-2 model.

#### 4.2.2. Rebar Strain

**Figure 10:**
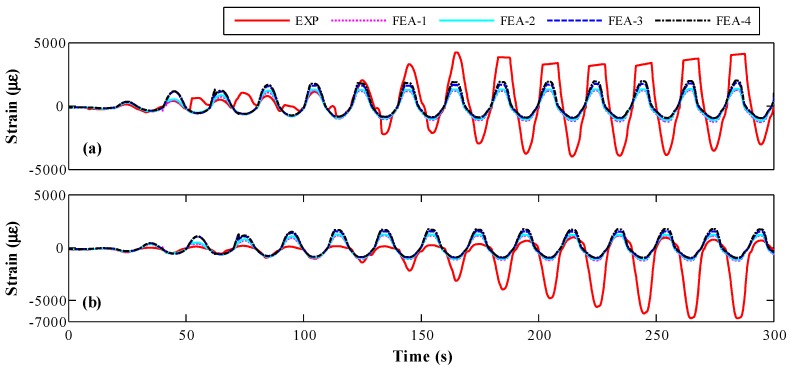
Strain time-history for different finite element model at different position along lateral loading direction: (**a**) −149 mm; and (**b**) 149 mm.

The strain time-histories of rebar derived from FE models and experiment are compared in Figure 10. The FE models have a good prediction in the early stage of strain time-history. As shown in Figure 10a,b, the experimental rebar strain mismatches with that of FE results after the second loading cycle, which was probably induced by the uniformity of load. The experimental amplitude of rebar strain clearly increased when the rebar yielded after eighth loading cycle, which might be caused by the uncoordinated deformation between concrete and rebar. Although the bond-slip effect was considered using concentrated plastic constitutive relationship in FE model, distributed plastic behavior still exists between rebar and concrete in plastic hinge zone. Since the part of rebar strain larger than yield strain did not generate any force based on the constitutive relationship of rebar, the actual differences in force borne by rebar between experiment and FE analysis decreased significantly.

#### 4.2.3. Cross-Section Moment

**Figure 11:**
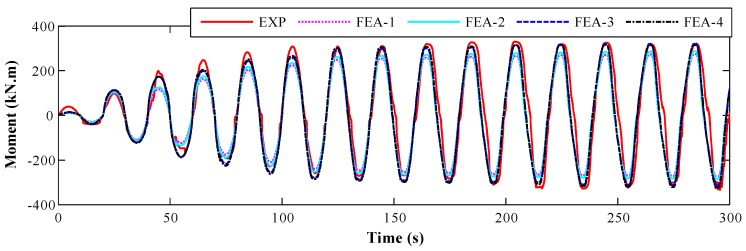
Time-history of the cross-section moment for experimental and analytical results.

To further validate the feasibility of SA-based concrete strength monitoring method, the typical moment time-history of cross-section A-A obtained from experiment and FE analysis was compared, as illustrated in Figure 11. Both the local concrete stress and rebar strain were adopted for the moment calculation, as reported in the literature [53]. It was found that the analytical moments increased with the increasing of *f_c_*. The results of FEA-3 and FEA-4 agree better with experimental moment than those of FEA-1 and FEA-2.

## 5. Error Analysis

To quantify the difference between the experimental and analytical result, a specific form of root-mean-square (RMS) error was adopted, as expressed in Equation (4)

(4) $$\varepsilon^{rms}=\frac{\left|x_{\exp}-x_{ana}\right|}{\left|x_{\exp}\right|}\times 100\%\;$$

where *x_exp_* and *x_ana_* are the amplitude of each cycle during cyclic loading for experimental and analytical curve, respectively.

### 5.1. Global Response

**Figure 12:**
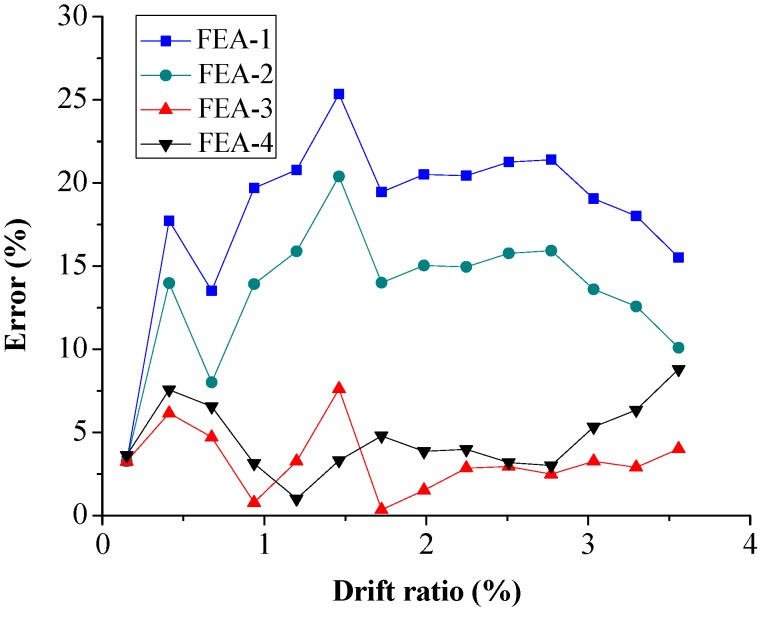
Root-mean-square error of lateral load.

Figure 12 shows the variation of the RMS error of lateral load with different concrete strength. The maximum errors of FEA-3 and FEA-4 are very close and less than 10%, which is much lower than those of FEA-1 and FEA-2. The maximum error of global response (i.e., lateral load) of simulations using peak stress of SA and estimated concrete strength are basically same. Thus, the assessment of concrete strength prediction cannot be effectively estimated using global response.

### 5.2. Local Response

**Figure 13:**
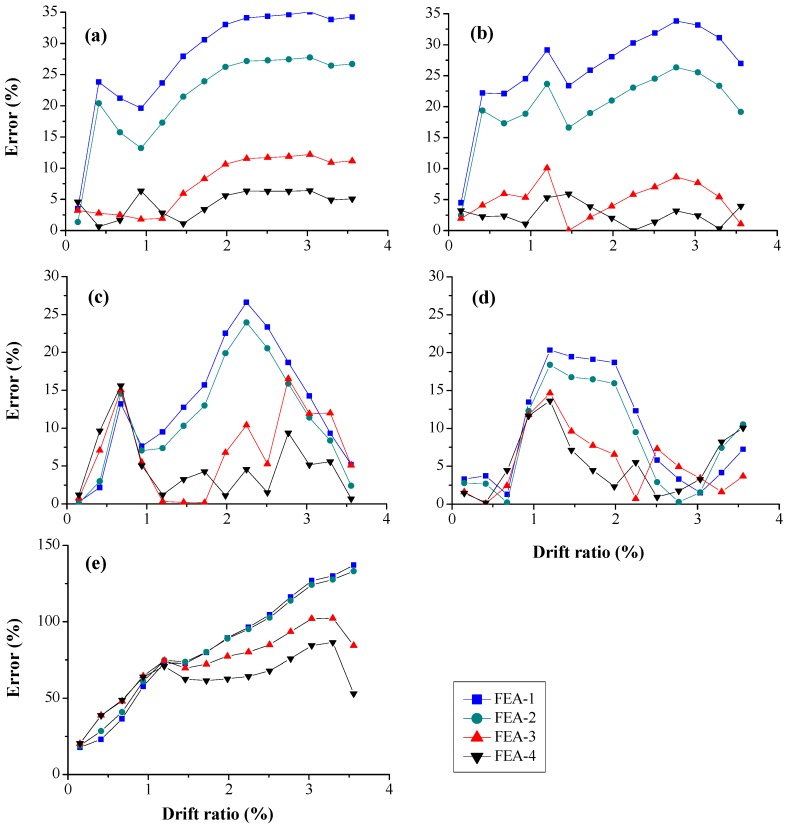
Root-mean-square error of concrete stress at the location of: (**a**) Point I; (**b**) Point V; (**c**) Point II, (**d**) Point IV; and (**e**) Point III.

The RMS error of concrete stress at five typical locations (Points I–V) is shown in Figure 13. For Points I and V (Figure 13a,b), the maximum error of FEA-4 is about 5%, which is lower than that of FEA-3. In addition, the errors of FEA-3 and FEA-4 are lower than those of FEA-1 and FEA-2. For Points II and IV (Figure 13c,d), the maximum RMS errors of FEA-3 and FEA-4 are about 15%, which are lower than those of FEA-1 and FEA-2 but larger than at Points I and V. The maximum RMS error of FEA-4 is lower than that of FEA-3, which is similar to those at Points I and V. For Point III (Figure 13c), the error is significantly larger than those obtained at the other points due to the complex stress status. The maximum RMS error of FEA-4 is not larger than that of FEA-3. Therefore, it can be concluded that the concrete stress in FE analysis using the SA stress-based estimated concrete strength agrees better with experimental result than with the strength obtained from the material test.

**Figure 14:**
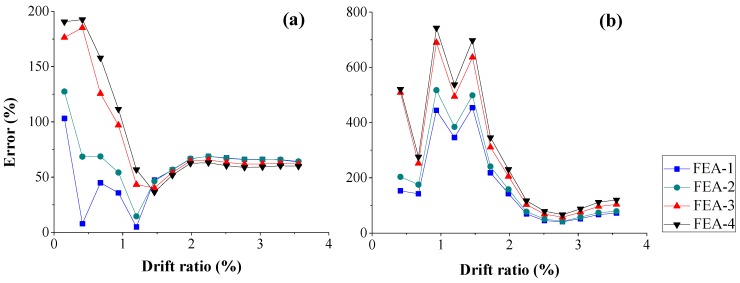
Root-mean-square error of rebar strain at different position along lateral loading direction: (**a**) −149 mm; and (**b**) 149 mm.

Figure 14 shows that the RMS error of the rebar strain using four different concrete strengths. The error increases with the increasing of concrete strength. This trend is opposite to lateral load or concrete strength, whereas the error is too large to be used for quantifying the accuracy of simulation.

**Figure 15:**
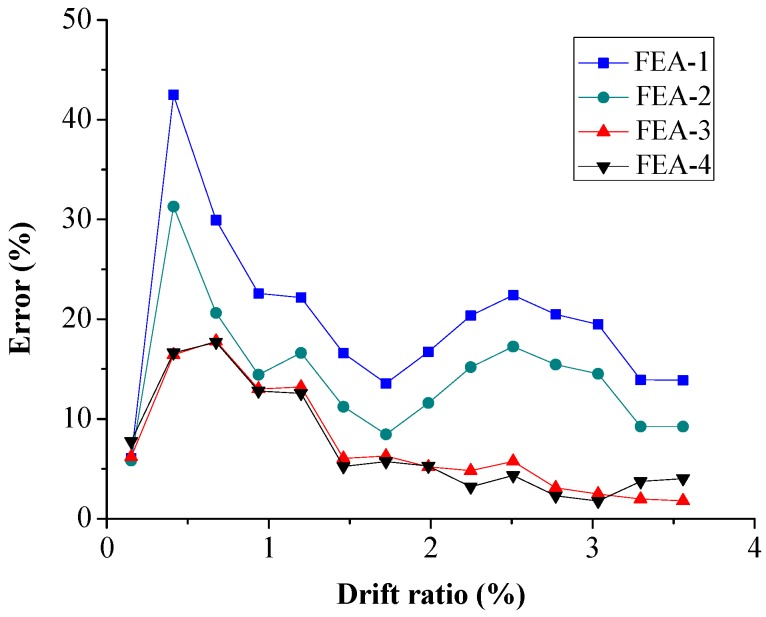
Root-mean-square error of cross-section moment.

The RMS error of cross-section moment is shown in Figure 15. The errors of FEA-3 and FEA-4 are smaller than those of FEA-1 and FEA-2. The maximum error of FEA-4 is about 20%, which is basically the same as that of FEA-3. That implies the index of cross-section moment is not suitable for concrete strength prediction.

## 6. Conclusions

This paper proposes the concrete strength validation of FE model using smart aggregate-based stress monitoring method. The concrete strengths obtained from material test, peak stress of SA, and estimated concrete strength based on SA stress were employed. The FE models were established, considering the load-end slip of rebar. To verify the proposed method, the global and local responses of the RC column and corresponding root-mean-square (RMS) error obtained from the experimental and numerical analysis were compared. The conclusions are summarized as follows.

The concrete stress of local response is a more sensitive index than global response and other local responses (rebar strain and cross-section moment) to identify the difference between experiment and FE simulation. The concrete compressive strength of the plastic hinge zone of RC structure obtained from standard specimens cast simultaneously provides a conservative estimate. The compressive strength in FE model can be validated using SA-based seismic stress monitoring method.

## Figures and Tables

**Table 1:** Parameters of concrete constitutive relationship.

Parameter	Lower Limit of Concrete Cube (FEA-1)	Average of Concrete Cube (FEA-2)	Monitored Peak Stress of SA (FEA-3)	Estimated Strength (FEA-4)
*f_cu_*	23.0	26.5	36.5	40.0
*f_cc_*	26.0	29.7	39.4	42.9
*f_uu_*	4.6	5.3	7.3	8.0
*f_uc_*	20.8	23.7	31.5	34.3
*ε_0u_*	0.00153	0.00158	0.00174	0.00179
*ε_0c_*	0.00173	0.00177	0.00188	0.00192
*ε_uu_*	0.0042	0.0038	0.0037	0.0035
*ε_uc_*	0.02	0.02	0.02	0.02

Note: The unit of *f_cu_, f_cc_, f_uu_* and *f_uc_* is MPa. The first subscripts “*c*“ and “*u*” stand for concrete strength and ultimate residual strength, respectively. The second subscripts “*c*” and “*u*” stand for confined and unconfined of concrete, respectively.

## References

[1] ACI Committee (2011). ACI 318-11: Building Code Requirements for Structural Concrete and Commentary.

[2] China Architecture & Building Press (2010). GB 50010-2010: The Code for Design of Concrete Structure, Ministry of Housing and Urban-Rural Development of the People’s Republic of China.

[3] Pereira N., Romão X. (2016). Assessment of the concrete strength in existing buildings using a finite population approach. Constr. Build. Mater..

[4] Bartlett M., Macgregor J.G. (1996). Statistical analysis of the compressive strength of concrete structures. ACI Mater. J..

[5] Drysdale R.G. (1973). Variation of concrete strength in existing buildings. Mag. Concr. Res..

[6] Stewart M.G. (1995). Workmanship and its influence on probabilistic models of concrete compressive strength. ACI Mater. J..

[7] Shang H.S., Song Y.P. (2006). Experimental study of strength and deformation of plain concrete under biaxial compression after freezing and thawing cycles. Cem. Concr. Res..

[8] Glasser F.P., Marchand J., Samson E. (2008). Durability of concrete—Degradation phenomena involving detrimental chemical reactions. Cem. Concr. Res..

[9] Khoury G.A. (2001). Effect of fire on concrete and concrete structures. Prog. Struct. Eng. Mater..

[10] Masi A., Vona M. (2009). Estimation of the in-situ concrete strength: Provisions of the European and Italian seismic codes and possible improvements. Eurocode 8 Perspectives from the Italian Standpoint Workshop.

[11] Breysse D., Martínez-Fernández J.L. (2014). Assessing concrete strength with rebound hammer: Review of key issues and ideas for more reliable conclusions. Mater. Struct..

[12] Alwash M., Breysse D., Sbartaï Z.M., Szilágyi K., Borosnyói A. (2017). Factors affecting the reliability of assessing the concrete strength by rebound hammer and cores. Constr. Build. Mater..

[13] Szilágyi K., Borosnyói A., Zsigovics I. (2011). Rebound surface hardness of concrete: Introduction of an empirical constitutive model. Constr. Build. Mater..

[14] Szilágyi K., Borosnyói A. (2009). 50 years of experience with the schmidt rebound hammer. Concr. Struct..

[15] Popovics J. (2005). Ultrasonic testing of concrete structures. Mater. Eval..

[16] Amini K., Jalalpour M., Delatte N. (2016). Advancing concrete strength prediction using non-destructive testing: Development and verification of a generalizable model. Constr. Build. Mater..

[17] Yıldırım H., Sengul O. (2011). Modulus of elasticity of substandard and normal concretes. Constr. Build. Mater..

[18] Qasrawi H.Y. (2000). Concrete strength by combined nondestructive methods simply and reliably predicted. Cem. Concr. Res..

[19] Teodoru G.V. (1989). The use of simultaneous nondestructive tests to predict the compressive strength of concrete. ACI Spec. Publ..

[20] ACI Committee (2003). ACI 228.1R-03: In-Place Methods to Estimate Concrete Strength.

[21] Li W., Kong Q., Ho S.C.M., Lim I., Mo Y.L., Song G. (2016). Feasibility study of using smart aggregates as embedded acoustic emission sensors for health monitoring of concrete structures. Smart Mater. Struct..

[22] Li Z., Qin L., Huang S. (2009). Embedded piezo-transducer in concrete for property diagnosis. J. Mater. Civ. Eng..

[23] Karaiskos G., Deraemaeker A., Aggelis D.G., Van Hemelrijck D. (2015). Monitoring of concrete structures using the ultrasonic pulse velocity method. Smart Mater. Struct..

[24] Dumoulin C., Karaiskos G., Carette J., Staquet S., Deraemaeker A. (2012). Monitoring of the ultrasonic P-wave velocity in early-age concrete with embedded piezoelectric transducers. Smart Mater. Struct..

[25] Song G., Gu H., Mo Y.L., Hsu T., Dhonde H., Zhu R.R.H. (2005). Health monitoring of a concrete structure using piezoceramic materials. Smart Structures and Materials 2005: Sensors and Smart Structures Technologies for Civil, Mechanical, and Aerospace Systems.

[26] Hou S., Zhang H.B., Ou J.P. (2012). A PZT-based smart aggregate for compressive seismic stress monitoring. Smart Mater. Struct..

[27] Kong Q.Z., Hou S., Ji Q., Mo Y.L., Song G. (2013). Very early age concrete hydration characterization monitoring using piezoceramic based smart aggregates. Smart Mater. Struct..

[28] Gu H., Song G., Dhonde H., Mo Y.L., Yan S. (2006). Concrete early-age strength monitoring using embedded piezoelectric transducers. Smart Mater. Struct..

[29] Song G., Gu H.C., Mo Y.L. (2008). Smart aggregates: Multi-functional sensors for concrete structures—A tutorial and a review. Smart Mater. Struct..

[30] Feng Q., Kong Q., Huo L., Song G. (2015). Crack detection and leakage monitoring on reinforced concrete pipe. Smart Mater. Struct..

[31] Yan S., Ma H., Li P., Song G., Wu J. (2017). Development and application of a structural health monitoring system based on wireless smart aggregates. Sensors.

[32] Yan S., Sun W., Song G., Gu H., Huo L.S., Liu B., Zhang Y.G. (2009). Health monitoring of reinforced concrete shear walls using smart aggregates. Smart Mater. Struct..

[33] Moslehy Y., Gu H., Belarbi A., Mo Y.L., Song G. (2010). Smart aggregate based damage detection of circular RC columns under cyclic combined loading. Smart Mater. Struct..

[34] Zhang J., Li Y., Du G., Song G. (2018). Damage detection of L-shaped concrete filled steel tube (L-CFST) columns under cyclic loading using embedded piezoceramic transducers. Sensors.

[35] Xu K., Deng Q., Cai L., Ho S., Song G. (2018). Damage detection of a concrete column subject to blast loads using embedded piezoceramic transducers. Sensors.

[36] Zou D.J., Li W.J., Liu T.J., Teng J. (2018). Two-dimensional water seepage monitoring in concrete structures using smart aggregates. Struct. Monitor. Maint..

[37] Zou D.J., Liu T.J., Huang Y.C., Zhang F.Y., Du C.C., Li B. (2014). Feasibility of water seepage monitoring in concrete with embedded smart aggregates by P-wave travel time measurement. Smart Mater. Struct..

[38] Liu T.J., Huang Y.C., Zou D.J., Teng J., Li B. (2013). Exploratory study on water seepage monitoring of concrete structures using piezoceramic based smart aggregates. Smart Mater. Struct..

[39] Song G., Olmi C., Gu H. (2007). An overheight vehicle-bridge collision monitoring system using piezoelectric transducers. Smart Mater. Struct..

[40] Hou S., Zhang H.B., Ou J.P. (2013). A PZT-based smart aggregate for seismic shear stress monitoring. Smart Mater. Struct..

[41] Zhang H.B., Hou S., Ou J.P. (2016). Smart aggregate-based seismic stress monitoring system using a specially designed charge amplifier. J. Intell. Mater. Syst. Struct..

[42] Hou S., Zhang H.B., Ou J.P. (2016). SA-based concrete seismic stress monitoring: A case study for normal strength concrete. Smart Mater. Struct..

[43] Zhang H.B., Hou S., Ou J.P. (2017). Feasibility of SA-based concrete seismic stress monitoring for high-strength concrete. J. Intell. Mater. Syst. Struct..

[44] Zhang H.B., Hou S., Ou J.P. (2018). Smart aggregates for monitoring stress in structural lightweight concrete. Measurement.

[45] Du G., Zhang J., Zhang J., Song G. (2017). Experimental study on stress monitoring of sand-filled steel tube during impact using piezoceramic smart aggregates. Sensors.

[46] Zhao J., Sritharan S. (2007). Modeling of strain penetration effects in fiber-based analysis of reinforced concrete structures. ACI Struct. J..

[47] Comite Euro-International du Beton (1993). CEB-FIP Model Code 1990: Design Code.

[48] Menegotto M., Pinto P.E. (1973). Method of analysis for cyclically loaded R.C. plane frames including changes in geometry and non-elastic behavior of elements under combined normal force and bending. Resistance and Ultimate Deformability of Structures Acted on by Well Defined Repeated Loads.

[49] Filippou F.C., Popov E.P., Bertero V.V. (1983). Effects of Bond Deterioration on Hysteretic Behavior of Reinforced Concrete Joints.

[50] Scott B.D., Park P., Priestley M.J.N. (1982). Stress-Strain behavior of concrete by overlapping hoops at low and high strain rates. ACI Struct. J..

[51] SouthEast University (2008). Concrete Structures: Volumne One, Principle of Concrete Structure Design.

[52] Park R., Priestley M.J.N., Gill W.D. (1982). Ductility of square-confined concrete columns. J. Struct. Div..

[53] Hou S., Zhang H.B., Ou J.P. (2018). SA-based concrete seismic stress monitoring: The influence of non-uniform stress fields. Eng. Struct..

[54] Hognestad E., Hanson N.W., McHenry D. (1955). Concrete Stress Distribution in Ultimate Strength Design. ACI J. Proc..

